# Mild behavioral impairment and its relation to tau pathology in preclinical Alzheimer’s disease

**DOI:** 10.1038/s41398-021-01206-z

**Published:** 2021-01-26

**Authors:** Maurits Johansson, Erik Stomrud, Philip S. Insel, Antoine Leuzy, Per Mårten Johansson, Ruben Smith, Zahinoor Ismail, Shorena Janelidze, Sebastian Palmqvist, Danielle van Westen, Niklas Mattsson-Carlgren, Oskar Hansson

**Affiliations:** 1 Clinical Memory Research Unit, Department of Clinical Sciences Malmö, Lund University, SUS, Malmö, Sweden; 2 Division of Clinical Sciences Helsingborg, Department of Clinical Sciences Lund, Lund University, Helsingborg, Sweden; 3 Department of Psychiatry, Helsingborg Hospital, Helsingborg, Sweden; 4 Memory Clinic, Skåne University Hospital, Malmö, Sweden; 5 Department of Psychiatry, University of California, San Francisco, CA USA; 6 Department of Internal Medicine, Sahlgrenska Academy, University of Gothenburg, Gothenburg, Sweden; 7 Department of Neurology, Skåne University Hospital, Lund, Sweden; 8 Departments of Psychiatry, Clinical Neurosciences, and Community Health Sciences, University of Calgary, 3280 Hospital Drive NW, Calgary, AB Canada; 9 Hotchkiss Brain Institute and O’Brien Institute for Public Health, University of Calgary, 3280 Hospital Drive NW, Calgary, AB Canada; 10 Diagnostic Radiology, Department of Clinical Sciences Lund, Lund University, Lund, Sweden; 11 Image and Function, Skåne University Hospital, Malmö, Sweden; 12 Wallenberg Center for Molecular Medicine, Lund University, Lund, Sweden

**Keywords:** Predictive markers, Psychiatric disorders

## Abstract

Mild behavioral impairment (MBI) is suggested as risk marker for neurodegenerative diseases, such as Alzheimer’s disease (AD). Recently, pathologic tau deposition in the brain has been shown closely related to clinical manifestations, such as cognitive deficits. Yet, associations between tau pathology and MBI have rarely been investigated. It is further debated if MBI precedes cognitive deficits in AD. Here, we explored potential mechanisms by which MBI is related to AD, this by studying associations between MBI and tau in preclinical AD. In all, 50 amyloid-β-positive cognitively unimpaired subjects (part of the BioFINDER-2 study) underwent MBI-checklist (MBI-C) to assess MBI, and the Alzheimer’s Disease Assessment Scale – Cognitive subscale (ADAS-Cog) delayed word recall (ADAS-DR) to assess episodic memory. Early tau pathology was determined using tau-PET ([^18^F]RO948 retention in entorhinal cortex/hippocampus) and cerebrospinal fluid (CSF) P-tau_181_. Regression models were used to test for associations. We found that higher tau-PET signal in the entorhinal cortex/hippocampus and CSF P-tau_181_ levels were associated with higher MBI-C scores (β = 0.010, SE = 0.003, *p* = 0.003 and β = 1.263, SE = 0.446, *p* = 0.007, respectively). When MBI-C and ADAS-DR were entered together in the regression models, tau-PET (β = 0.009, *p* = 0.009) and CSF P-tau_181_ (β *=* 0.408, *p* = 0.006) were predicted by MBI-C, but not ADAS-DR. We conclude that in preclinical AD, MBI is associated with tau independently from memory deficits. This denotes MBI as an important early clinical manifestation related to tau pathology in AD.

## Introduction

Alzheimer’s disease (AD) is the leading cause of dementia, affecting tens of millions of people worldwide^1^. Clinically, it is characterized by multifaceted symptoms including both cognitive deterioration and advancing neuropsychiatric or behavioral manifestations, ultimately leading to severe functional disability^2^. Neuropathological hallmarks of AD include amyloid-β (Aβ) plaques and neurofibrillary tangles comprised of hyperphosphorylated tau^2^.

Lately, positron emission tomography (PET) studies have suggested that abnormal tau deposition rarely occur without presence of abnormal Aβ deposition, and further that tau is more strongly related, than Aβ, to the onset of cognitive decline and neurodegeneration^3–5^. Hence, tau pathology can serve as a good marker of disease mechanisms involved in the early manifestations of AD. Yet, few studies have explored the link between regional cerebral tau pathology and the behavioral manifestations in this disease^6–9^.

Mild behavioral impairment (MBI) is a fairly novel concept, and is a neurobehavioral syndrome characterized by emergent, persistent, and diverse neuropsychiatric or behavioral symptoms late in life^10^. The syndrome is shown both detectable and prevalent in community and clinical samples^11–14^. The Alzheimer’s Association International Society to Advance Alzheimer’s Research and Treatment (ISTAART-AA) MBI criteria were developed to provide a standardized framework to explore these neuropsychiatric or behavioral symptoms as early manifestations of neurodegenerative disease and further to detect subjects at increased risk of dementia. The criteria also emphasize that MBI can occur in advance of, in concert with, or following mild cognitive impairment (MCI)^10^. Yet, it still remains to be demonstrated, whether MBI precedes or follows cognitive deficits in biomarker confirmed early AD, and whether MBI is associated with tau pathology at this preclinical phase.

In the present study we aimed to explore the cross-sectional association between MBI and biomarkers of tau pathology in preclinical AD and to compare associations with tau for MBI and episodic memory deficit.

## Materials and methods

### Study sample

The sample encompassed participants from three cohorts in the prospective and longitudinal Swedish BioFINDER−2 (BF-2) study (clinical trial no. NCT03174938), which were recruited from two centers in southern Sweden. Only cognitively unimpaired (CU) Aβ-positive subjects (*n* = 50),), representing preclinical Alzheimer’s pathological change or preclinical AD according to the NIA-AA research framework^2^, were included in the present study. Definition of Aβ-positivity is provided under section Cerebrospinal fluid biomarkers. Of these, 25 were recruited as controls and 25 as subjects with subjective cognitive decline (SCD). Controls were recruited from the BF-2 cohort A (aged 40–65 years) and B (aged 66–100 years), which represented neurologically and cognitively healthy controls. The SCD subjects were recruited from the BF-2 cohort C which includes subjects with either SCD or MCI. Classification for having SCD or MCI were based on performance on a neuropsychological test battery. This classification procedure as well as inclusion criteria for the separate cohorts are described in detail in the supplement (Supplementary eMethods). In agreement with the National Institute on Aging - Alzheimer’s Association (NIA-AA) research framework, subjects with SCD were analyzed together with the controls as CU^2^.

### Standard protocol approvals, registrations, and patient consents

The Regional Ethical Review Board in Lund, Sweden approved the study. All participants gave their written informed consent. Approval for PET imaging was obtained from the Swedish Medicines and Products Agency, and the local Radiation Safety Committee at Skåne University Hospital, Sweden.

### Clinical assessments

The global burden of MBI was measured using the Swedish version of the MBI checklist (MBI-C: www.MBItest.org)^15^. The MBI-C was developed to capture MBI in accordance with the ISTAART-AA MBI criteria, with language suitable for functionally independent community dwelling older adults. Importantly, the MBI-C mandates that symptoms have emerged late in life and persist for at least 6 months duration. The MBI-C has been validated in population-based samples^16^, as well as in samples of patients with SCD^12^ and MCI^11^. The MBI-C covers 34 items, representing five domains: (1) decreased drive and motivation (apathy, comprising 6 items, range 0–18 points), (2) affective dysregulation (mood and anxiety symptoms, comprising 6 items, range 0–18 points), (3) impulse dyscontrol (agitation, impulsivity, and abnormal reward salience, comprising 12 items, range 0–36 points), (4) social inappropriateness (impaired social cognition, comprising 5 items, range 0–15 points), and (5) abnormal perception and thought content (psychotic symptoms, comprising 5 items, range 0–15 points). Each question is answered with “No” (0 points) or “Yes” depending on if the actual symptoms have persisted for at least 6 months (continuously or intermittently) and represent a clear alteration from that person’s normal behavior. Items answered “Yes” are followed by a severity rating of either 1 point = mild, 2 points = moderate, or 3 points = severe. A MBI-C total score (range 0–102 points) is calculated by summing the scores of each item. Using a similar approach total scores for each domain can be generated. Subjects with >20% missing item responses (*n* = 1) were excluded prior to enrollment in this study. Eight (16.0%) subjects had one or two missing MBI-C responses out of 34 items in total. In the whole data set, 10 (0.6%) item responses were missing out of 1700. The “drive and motivation” domain item 6 was missing in 6 cases (12.0%) and in the remaining items <2.0% were missing. In our study, we used the informant-rated version of MBI-C, completed on an average of 0.8 months [2.1 SD] from baseline and 0.7 months [2.1 SD] before tau-PET with [^18^F]RO948.

Episodic memory impairment was assessed using the Alzheimer’s Disease Assessment Scale – Cognitive subscale (ADAS-Cog) task “10 word Delayed Recall” (ADAS-DR) (measured on a scale of 0–10, where a score of 0 indicates no memory impairment and 10 indicates severe impairment of short-term memory)^17,18^.

### Tau-PET scanning and processing

[^18^F]RO948-PET was performed on a digital GE Discovery MI scanner, 70–90 min post-injection. Standardized uptake value ratio (SUVR) images were created using the inferior cerebellar cortex as reference region^19^. Complete PET details are described elsewhere^20^.

In order to capture brain areas affected by tau deposition over the course of AD, three composite FreeSurfer-based regions-of-interest (ROI) were created according to the Braak tau pathology staging scheme^21^. These include region I-II (the entorhinal cortex and hippocampus), region III-IV (parahippocampal cortices, fusiform cortices, amygdala, as well as the inferior and the middle temporal cortices), and region V-VI (widespread neocortical areas)^22^. In a secondary analysis, regional associations between MBI-C total scores and [^18^F]RO948 SUVR were assessed using voxel-wise multiple regression models, including age, sex, education, and white matter volume as covariates, as implemented in SPM12 (https://www.fil.ion.ucl.ac.uk/spm/software/spm12/).

### MRI acquisition and processing

High‐resolution anatomical T1-weighted MRI images were obtained using a Siemens-3T MAGNETOM Prisma scanner for PET image co-registration and template normalization, as described elsewhere^20^. A T2-weighted fluid-attenuated inversion recovery (FLAIR) sequences was also performed and used to obtain a volume measure of global white matter lesions (WML) volume, extracted using the LST toolbox for automated lesion prediction^23^, as implemented in SPM12.

### Cerebrospinal fluid biomarkers

To acquire cerebrospinal fluid (CSF), lumbar puncture was performed at baseline for the subjects in cohort A and B, while for subjects in cohort C this was performed within 3 months prior to baseline. CSF levels of tau phosphorylated at threonine 181 (P-tau_181_) were obtained using Innotest^®^ immunoassay (Fujirebio; Gent, Belgium). Aβ_42_ and Aβ_40_ were determined using Meso Scale Discovery immunoassays (MSD; Rockville, MD, USA)^24^. Aβ-positivity in this study was defined by Aβ_42_/Aβ_40_ < 0.752 (cut-off obtained using Gaussian mixture modeling^25^). All CSF analyses were performed at the Clinical Neurochemistry Laboratory, Sahlgrenska University Hospital, Mölndal, Sweden.

### Statistical analyses

First, items with missing scores were dropped, with MBI-C total and domain-specific scores calculated by summing up available item responses. However, for robustness we also handled missing item responses (10 data points out of 1700 for the included subjects, one subject was excluded from the study due to >20% missing items) using a single imputation procedure, as implemented via the “aregImpute” function in the Hmisc package in R. This function uses additive regression, bootstrapping, and predictive mean matching to impute missing values. Due to very low response frequencies in the higher MBI-C item response classes (severity rating 2–3), which did not meet the minimum sample size requirements of the “aregImpute” function, classes had to be collapsed for several items. The results from the non-imputed data set are reported as the main findings, while outcomes on the imputed data set are provided in the supplement (Supplementary Tables 1 and 2).

Second, we performed multivariate linear regression analyses to evaluate associations between MBI and tau biomarkers. MBI-C total was used as the independent variable, while tau-PET SUVR (in the various Braak stages) or CSF P-tau_181_ were entered as dependent variables in separate models. In addition to age, sex, and educational level models were adjusted for WML burden due their prevalence as a comorbidity in AD and their relation to some neuropsychiatric symptoms, e.g., apathy^26,27^. Model effect estimates are reported as β-values, variance as standard error of the mean (SE) and statistical significance as *p*-values. In a secondary analysis we used a voxel-based approach to investigate the relationship between MBI and tau-PET SUVR.

Third, when an association was seen between MBI-C total score and tau-PET or CSF P-tau_181_, we conducted similar regression models with ADAS-DR as the independent variable. Additionally, we explored if MBI and episodic memory deficit were independently associated with tau pathology or confounded each other in a combined model where both MBI-C and ADAS-DR were entered as independent variables. In these combined models, MBI-C and ADAS-DR were standardized using *z*-scores.

Lastly, to explore how tau pathology is related to the underlying MBI-C domains, the total score for each domain was entered as the independent variable in similar separate models, as described above.

For all statistical tests, a significance threshold of *p* < 0.05 (two-sided) was used. Adjustment for multiple comparisons has not been made given the explorative approach of this study. Model assumptions for linear regression were checked by evaluating normality and homoscedasticity of residuals versus fitted values. All analyses were performed using R version 3.6.1.

## Results

### Demographics and clinical characteristics

Demographic and clinical characteristics are displayed in Table 1. Mean age across all subjects was 72.3 years (9.7 SD), 50% were females and the mean Mini-Mental State Examination (MMSE) score was 28.8 (1.3 SD). Mean number of years in education was 12.0 years (3.6 SD). Mean MBI-C total score was 4.0 (6.5 SD). The MBI-C domains social inappropriateness and, abnormal perception and thought content displayed lower mean scores (0.2 [0.8 SD] and 0.1 [0.4 SD], respectively) compared to the other domains.

**Table 1:** Sample characteristics.

Characteristic (*n* = 50)			
Demographics			
Female sex, *n* (%)	25	(50.0)	–
Age (year), mean (SD), min–max	72.3	(9.7)	44–88
Education (year), mean (SD), min–max	12.0	(3.6)	7–21
Clinical assessments			
ADAS-DR, mean (SD), min–max	3.4	(2.0)	0–8
MMSE, median (IQR), min–max	29.0	(2.0)	25–30
MBI-C total, median (IQR), min–max	6.0	(12.0)	0–44
MBI-C Motivation, median (IQR), min–max	1.0	(4.0)	0–15
MBI-C Mood, median (IQR), min–max	2.0	(4.0)	0–15
MBI-C Impulse dyscontrol, median (IQR), min–max	2.0	(4.0)	0–15
MBI-C Social inappropriateness, median (IQR), min–max	<0.1	(1.0)	0–7
MBI-C Perception, median (IQR), min–max	<0.1	(<0.1)	0–9
Pathology measurements			
APOE4/E4 carrier status, *n* (%)	4.0	(8.0)	–
SUVR - Braak region I-II, mean (SD), min–max	1.2	(0.2)	0.75–1.6
WMLvol, median (IQR), min–max	4.2	(15.6)	0.0–44.7
CSF Aβ_42_/Aβ_40_ ratio, mean (SD), min–max	0.6	(0.1)	0.3–0.7
CSF P-tau_181_, mean (SD), min–max	61	(21)	28–159

Demographic and clinical characteristics of the 50 CU-Aβ-positive subjects. Aβ-positivity was defined by previously defined cut-off (CSF Aβ_42_/Aβ_40_ ratio <0.752). Continuous normally distributed variables are presented with mean, SD, and minimum-maximum values (min–max), while non-normally distributed data is presented with medians, IQR, and min–max.

*Aβ* amyloid-β, *ADAS-DR* ADAS-Cog delayed recall, *APOE* apolipoprotein E, *CU* cognitively unimpaired, *CSF* cerebrospinal fluid, *IQR* interquartile range, *MBI-C* mild behavioral impairment – checklist, *MMSE* Mini-Mental State Examination, *p* *p*-value, *P-tau* phosphorylated tau, *SD* standard deviation, *SUVR* standard uptake value ratio, *WMLvol* volume of white matter lesions.

### Associations between MBI-C total score and tau pathology

**Fig. 1:**
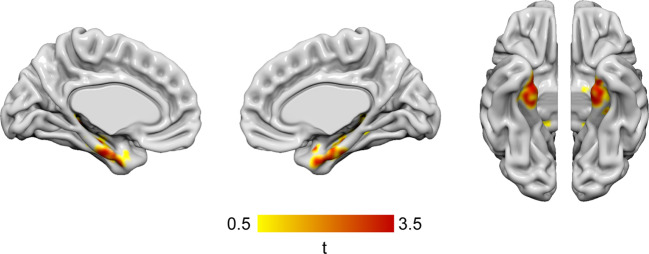
Whole-brain voxel-based analysis between MBI-C scores and tau-PET in cognitively unimpaired Aβ-positive subjects. Voxel-based associations between mild behavioral impairment – checklist (MBI-C) total scores and [^18^F]RO948-PET standard uptake value ratio (SUVR) in 50 Aβ-positive cognitively unimpaired subjects. Statistical significance was determined using an extent threshold of 50 voxels. Models were adjusted for age, sex, years of education, and volume of white matter lesions. Correction for multiple testing was applied to parametric images using false discovery rate (FDR) *p* < 0.05. Associations were confined to the entorhinal cortex and hippocampus, as well as to a smaller extent the anterior fusiform gyrus.

The MBI-C total score was significantly associated with tau-PET SUVR in the Braak I-II ROI (β = 0.010, SE = 0.003, *p* = 0.003) as well as with CSF P-tau_181_ levels (β = 1.263, SE = 0.446, *p* = 0.007) (Table 2). MBI-C was not predictive of tau deposition in the two other composite regions (Braak III-IV ROI: β = 0.003, SE = 0.005, *p* = 0.559; Braak V-VI ROI: β = 0.002, SE = 0.003, *p* = 0.510). Similar results were obtained using the imputed data set (Supplementary Table 1). The whole-brain voxel-wise analysis revealed significant relationship between MBI-C and tau deposition in the entorhinal cortex and hippocampus, as well as to a lesser degree in the anterior fusiform gyrus (Fig. 1).

**Table 2:** MBI-C and ADAS-DR as predictors of tau pathology.

Model	β	SE	*p*	^4^Δ*R*^2^	^3^*R*^2^
^1^Braak I-II ~ MBI-C	0.010	0.003	0.003	0.160	0.300
^1^Braak I-II ~ ADAS-DR	0.025	0.013	0.065	0.065	0.204
^2^Braak I-II ~ MBI-C (+ADAS-DR)	0.009	0.003	0.009	0.117	0.322
^2^Braak I-II ~ ADAS-DR (+MBI-C)	0.015	0.013	0.242	0.022	0.322
^1^P-tau_181_ ~ MBI-C	1.263	0.446	0.007	0.139	0.236
^1^P-tau_181_ ~ ADAS-DR	0.299	1.884	0.875	0.001	0.098
^2^P-tau_181_ ~ MBI-C (+ADAS-DR)	1.347	0.468	0.006	0.146	0.244
^2^P-tau_181_ ~ ADAS-DR (+MBI-C)	−1.163	1.817	0.525	0.008	0.244

Multivariate linear regression analyses on the 50 CU Aβ-positive subjects. Models were adjusted for age, sex, education, and WML volume. [^18^F]RO948-PET SUVR in regions representing Braak stage I-II (the entorhinal cortex and hippocampus) or CSF P-tau_181_ were entered as the continuous dependent variable in the separate models. MBI-C and ADAS-DR scores were standardized (*z*-scores). ^1^In individual models MBI-C and ADAS-DR, respectively, were entered as the independent variable. ^2^In combined models both MBI-C and ADAS-DR were entered as predictor and a covariate to be adjusted for, respectively. ^3^*R*^2^ for the complete model. ^4^Change in *R*^2^ when adding MBI or ADAS-DR to models initially only including the covariates.

*β* beta coefficient, *Aβ* amyloid-β, *ADAS-DR* ADAS-Cog delayed recall, *CU* cognitively unimpaired, *MBI-C* mild behavioral impairment – checklist, *p* *p*-value, *P-tau*_*181*_ phosphorylated tau 181, *R*^2^ the coefficient of determination, *SE* standard error, *SUVR* standard uptake value ratio.

### MBI and memory deficit as predictors of tau pathology

The ADAS-DR scores predicted tau-PET signal in the Braak stage I-II ROI only at a trend level (β=0.025, SE = 0.013, *p* = 0.065) (Table 2). In the combined model, where both MBI-C and ADAS-DR were entered simultaneously in order to control for each other, only MBI-C remained associated with tau-PET (β = 0.009, SE = 0.003, *p* = 0.009). Similar results were found for CSF P-tau_181_ in the combined models, for which only MBI remained associated with CSF P-tau_181_ (β = 0.408, SE = 0.142, *p* = 0.006). The imputed data set rendered comparable results (Supplementary Table 1).

### Associations between MBI-C domains and tau pathology

The MBI-C domains affective dysregulation (β=0.025, SE = 0.009, *p* = 0.006) and impulse dyscontrol (β = 2.347, SE = 0.907, *p* = 0.013) were associated with tau-PET SUVR in the Braak I-II ROI as well as CSF P-tau_181_ levels (Table 3). The other MBI-C domains did not display any associations with tau pathology. Also here, the imputed data set provided similar results (Supplementary Table 2).

**Table 3:** The different MBI-C domains as predictors of tau deposition in Braak region I-II.

Model	β	SE	*p*	^2^Δ*R*^2^	^1^*R*^2^
Braak I-II ~ MBI-C Drive	0.030	0.017	0.088	0.055	0.195
Braak I-II ~ MBI-C Affective	0.025	0.009	0.006	0.138	0.277
Braak I-II ~ MBI-C Control	0.020	0.006	0.003	0.158	0.298
Braak I-II ~ MBI-C Social	0.028	0.031	0.134	0.015	0.155
Braak I-II ~ MBI-C Perception	0.011	0.052	0.835	0.001	0.140
P-tau_181_ ~ MBI-C Drive	4.181	2.400	0.088	0.058	0.156
P-tau_181_ ~ MBI-C Affective	3.302	0.268	0.008	0.136	0.233
P-tau_181_ ~ MBI-C Control	2.347	0.907	0.013	0.119	0.216
P-tau_181_ ~ MBI-C Social	2.897	4.340	0.508	0.009	0.106
P-tau_181_ ~ MBI-C Perception	2.880	7.146	0.689	0.003	0.101

Multivariate linear regression analyses in 50 CU Aβ-positive subjects, to investigate the association between tau deposition in Braak stage I-II or using CSF P-tau_181_, and the five different MBI-C domains (Drive and motivation [Drive], Affective regulation [Affective], Impulse dyscontrol [Control], Social inappropriateness [Social], Perception and thought [Perception]). Models were adjusted for age, sex, education, and WML volume. [^18^F]RO948-PET SUVR in Braak stage I-II or CSF P-tau_181_ was entered as a continuous dependent variable. Continuous MBI-C domain scores, respectively, were entered as the independent variable. ^1^*R*^2^ for the complete model. ^2^Change in *R*^2^ when adding MBI to models initially only including the covariates.

*β* beta coefficient, *Aβ* amyloid-β, *CU* cognitively unimpaired, *MBI-C* mild behavioral impairment – checklist, *p* *p*-value, *P-tau*_*18*1_ phosphorylated tau 181, *R*^2^ the coefficient of determination, *SE* standard error, *SUVR* standard uptake value ratio.

## Discussion

In this cross-sectional study, which is one of the first to focus on subjects with preclinical AD, we found that MBI scores, but not episodic memory impairment, were independently associated with early tau pathology determined using either CSF P-tau_181_ or tau-PET. Tau-PET associations were confined to the Braak I-II regions (the entorhinal cortex and hippocampus), according to both ROI and voxel-based approaches.

### MBI and regional tau deposition

In contrast to our findings, a recent study addressing the association between MBI and AD biomarkers (amyloid and tau-PET) in CU subjects found that MBI scores were associated with global and striatal amyloid-PET signal, but not with tau-PET^6^. This study, however, also included Aβ-negative cases; as abnormal tau PET is essentially only seen in the context of Aβ-positivity^4,5^, this may have resulted in the absence of an association between MBI and tau PET or in a study that was underpowered to detect such an association. More in line with our results, another recent study found an association between tau deposition and multiple behavioral features (including personality traits such as neuroticism and openness, apathy, depression, lifetime cognitive activity) in a cohort of CU older adults at increased risk of AD due to a positive family history of sporadic disease^8^. Similar to our findings, tau-PET signal in the entorhinal cortex showed the strongest association to behavioral features.

Disturbances in drive and emotions in AD are often referred to as manifestations of frontal lobe pathology, wherefore an association with temporal tau deposition could be considered a somewhat unexpected finding in its relation to neuropsychiatric symptoms. Yet, most pathological studies displaying such frontal associations have been conducted on MCI and AD dementia samples, not on CU samples^27,28^. Some studies focusing on cerebral atrophy have instead strengthened the role of the temporal lobe pathology in neuropsychiatric and behavioral symptoms, such as apathy, among CU subjects^29,30^. Bridging the gap, we recently reported similar results among CU and MCI cases, arguing for a higher level of apathy to be associated with atrophy predominately in the temporal lobe, and also to a lesser extent the frontal lobe^26^. Taken together, these initial CU studies, using MRI and tau-PET, point to core AD pathologies in the temporal lobe to play important roles in the early development of neuropsychiatric or behavioral symptoms.

### MBI versus memory deficit in relation to tau pathology

Memory loss is often referred to as one of the main findings in the early clinical stages of AD^31^. Hypothetically, this could mediate the association between MBI scores and tau in the entorhinal cortex and the hippocampus. For instance, awareness over emergent memory deficits could give rise to anxiety or other affective disturbances over having a potential underlying neurodegenerative disease. Our regression models, however, using both tau-PET and CSF P-tau_181_, showed that episodic memory impairment was not associated with tau pathology. Furthermore, the association between MBI and tau deposition survived correction for memory performance. Taken together, this indicates that the association between MBI and tau is independent from memory deficits and positions MBI as an important and early clinical manifestation of tau pathology in AD. Additionally, the fact that MBI associated with tau but not memory deficits in a cohort of CU individuals lends support to the ISTAART-AA MBI criteria indicating that MBI can precede MCI^10^. In this respect, 30% of the CU participants in the National Alzheimer Coordinating Center (NACC) data set that later developed AD dementia displayed neuropsychiatric or behavioral symptoms before the diagnosis of MCI^32^. Combined, these studies indicate that MBI has the potential to aid in early risk assessments of underlying neuropathology.

Mechanistically, the interplay between MBI and cognitive symptoms in preclinical AD (i.e., Aβ-positive CU) is complex. Our findings showed associations between MBI and regional tau deposition in the entorhinal cortex and hippocampus, regions shown to be affected by tau pathology early on in the course of AD^21^. Therefore, confinement of associations in these regions align well with our use of a CU sample. Nonetheless, these are structures not primarily ascribed roles in emotion and behavior. The hippocampus, however, is highly interconnected with the amygdala, a region important for emotional processing. Hippocampus and amygdala have also been suggested to interact synergistically during the process of long-term memory formation^33,34^. Together, they also display widespread connections to other important parts of the brain related to both cognitive and emotional functioning, including the frontal cortex^33,34^. Altogether, it could be hypothesized that early tau deposition in the hippocampus has an indirect but close effect on emotions via disruption of the emotional brain network.

### The MBI domains and their associations with tau pathology

Only the MBI-C domains of affective regulation and impulse dyscontrol were shown to be related to tau pathology. The former consists of items covering both anxiety and depressive symptoms, while the later encompasses irritability, agitation, and aggression^15^. Thus, we find these results to be in concordance with the findings by Gatchel et al. reporting depressive symptoms related to temporal tau deposition^35^ and by Ramakers et al. reporting anxiety associated with CSF total tau^36^. Impulse dyscontrol and agitation have lately attracted research interest as potential early markers of dementia in mid and late life^37^. In support of our results, agitation and aggression have previously been linked to core AD CSF biomarkers^38^. Further highlighting the role of affective disorders and impulse dyscontrol, within the earliest stages of neurodegenerative disease, both depression and irritability have been demonstrated to be some of the most common manifestations preceding cognitive decline^32^. Nonetheless, more studies are needed to fully unravel the link between these constructs and early AD pathology.

### Limitations and strengths

First, the cross-sectional nature of this study renders it difficult to fully explore the temporal order of appearance of neuropsychiatric symptoms and cognitive deficits. Second, the study sample size is somewhat modest, which needs to be taken into account when the results are interpreted. Adding to this, the total scores for some MBI domains were quite low, which could have reduced the statistical power for these specific analyses. Third, ADAS-DR might not fully detect the most subtle changes in memory performance among otherwise cognitively healthy subjects. Moreover, as we only included Aβ-positive CU subjects, these findings do not necessarily apply to CU subjects in general. Yet, this is also an important strength to the study, since we investigated subjects with a particularly high risk of developing cognitive deficits, thus also those most appropriate for future AD secondary prevention trials. Additional strengths of this study include that we report on a well-characterized sample of subjects with preclinical AD where cerebrovascular disease has been controlled for. Another is the reporting on both unimputed and imputed data, as well as the fact that similar results were obtained using two different markers for early tau pathology (CSF P-tau and tau-PET signal in Braak 1-2 ROI).

## Conclusion

These findings denote MBI as an important early clinical manifestation associated with tau pathology in preclinical AD, which further could have implications for clinical care and AD clinical trials. Future studies should aim to validate these findings in larger samples and preferably with the use of longitudinal data.

## Supplementary information

Supplementary information.

## Data Availability

The code that support the findings of this study will be shared by request from a qualified academic investigator.
