# Supplementary material for: Mild behavioral impairment and its relation to tau pathology in preclinical Alzheimer’s disease
Source: Transl Psychiatry. 2021 Jan 26;11:76. doi: 10.1038/s41398-021-01206-z (PMC7838407; doi:10.1038/s41398-021-01206-z)
Supplement: Supplementary file 1 — Supplementary information. [file 41398_2021_1206_MOESM1_ESM.docx]

**Supplementary information**

# Mild Behavioral Impairment and its relation to tau pathology in preclinical Alzheimer’s disease

Maurits Johansson, MD; Erik Stomrud, MD, PhD; Philip S. Insel, MS; Antoine Leuzy, PhD; Per Mårten Johansson, MD, PhD; Ruben Smith, MD, PhD; Zahinoor Ismail, MD; Shorena Janelidze, PhD; Sebastian Palmqvist, MD, PhD; Danielle van Westen, MD, PhD; Niklas Mattsson-Carlgren, MD, PhD; Oskar Hansson, MD, PhD.

Supplementary material

1. eMethods – Study sample. Page 2.
2. Table 1 – MBI-C and ADAS-DR as predictors of tau pathology (imputed data). Page 4
3. Table 2 – MBI-C domains as predictors of tau deposition in Braak region I-II (imputed data). Page 5.
4. Supplementary references (eMethods). Page 6.

**Supplementary eMethods – Study sample**

The sample encompassed participants from three cohorts in the prospective and longitudinal Swedish BioFINDER-2 (BF-2) study (clinical trial no. NCT03174938), which were recruited from two centers in southern Sweden. Only cognitively unimpaired (CU) Aβ-positive (definition of Aβ-positivity is provided below under section 2.6) subjects were included (n=50) in the present study, representing preclinical Alzheimer’s pathological change or preclinical AD^1^. Of these, 25 were recruited as controls and 25 as subjects with subjective cognitive decline (SCD). Controls were recruited from the BF-2 cohorts A and B, which represented neurologically and cognitively healthy controls. The inclusion criteria were: i) age 40-65 years (cohort A) and age 66-100 years (cohort B); ii) absence of cognitive symptoms as evaluated by a physician specialized in cognitive disorders; iii) Mini Mental State Examination (MMSE) score 27-30 (cohort A) or 26-30 (cohort B); iv) did not fulfill the criteria for mild or major neurocognitive disorder (MCI or dementia) according to DSM-5 ^2^; and v) fluency in Swedish. The SCD subjects were recruited from the BF-2 cohort C which includes subjects with SCD or MCI. Inclusion criteria were: i) age 40-100 years; ii) referred to one of the memory clinics because of cognitive symptoms; iii) MMSE score 24-30; iv) did not meet the criteria for any kind of dementia (major neurocognitive disorder) according to DSM-5^2^, v) fluency in Swedish.

If subjects in cohort C performed worse than -1.5 SD in any cognitive domain, using a neuropsychological test battery, according to age and education stratified test norms, they were classified as having MCI. The neuropsychological battery covered the domains attention (Trail Making Test A and Symbol Digit Modalities Test), executive function (Trail Making B and A Quick Test of cognitive speed [AQT]), verbal ability (verbal fluency animals and the 15 word short version of the Boston Naming Test), memory (immediate and delayed recall from the Alzheimer’s Disease Assessment Scale [ADAS]), and visuospatial function (incomplete letters and cube analysis from the Visual Object and Space Perception battery [VOSP]). Subjects not classified as MCI were considered to have SCD.

In agreement with National Institute on Aging - Alzheimer’s Association (NIA-AA) research framework, subjects with SCD were analyzed together with the controls as CU ^1^.

**Supplementary Table 1.** MBI-C and ADAS-DR as predictors of tau pathology (imputed data).

| **Model** | **β** | **S.E** | **p** | ^§^**ΔR^2^** | ^‡^**R^2^** |
| --- | --- | --- | --- | --- | --- |
| ^*^Braak I-II ~ MBI-C | 0.010 | 0.003 | **0.009** | 0.125 | 0.265 |
| ^*^Braak I-II ~ ADAS-DR | 0.025 | 0.013 | 0.065 | 0.065 | 0.204 |
| ^†^Braak I-II ~ MBI-C (+ ADAS-DR) | 0.009 | 0.004 | **0.025** | 0.089 | 0.293 |
| ^†^Braak I-II ~ ADAS-DR (+ MBI-C) | 0.017 | 0.013 | 0.198 | 0.028 | 0.293 |
| ^*^P-tau_181_ ~ MBI-C | 1.463 | 0.504 | **0.006** | 0.145 | 0.242 |
| ^*^P-tau_181_ ~ ADAS-DR | 0.299 | 1.884 | 0.875 | 0.001 | 0.098 |
| ^†^P-tau_181_ ~ MBI-C (+ ADAS-DR) | 1.548 | 0.527 | **0.005** | 0.151 | 0.249 |
| ^†^P-tau_181_ ~ ADAS-DR (+ MBI-C) | -0.110 | 1.803 | 0.545 | 0.007 | 0.249 |

Multivariate linear regression analyses on the 50 CU Aβ-positive subjects using single imputed data for missing MBI-C item responses. Models were adjusted for age, sex, education and WML volume. ^18^F-RO948-PET SUVR in regions representing Braak stage I-II (the entorhinal cortex and hippocampus) or CSF P-tau_181_ were entered as the continuous dependent variable in the separate models. MBI-C and ADAS-DR scores were standardized (z-scores). ^*^In individual models MBI-C and ADAS-DR, respectively, were entered as the dependent variable. ^†^In combined models both MBI-C and ADAS-DR were entered as predictor and a covariate to be adjusted for, respectively. ^‡^R^2^ for the complete model. ^§^Change in R^2^ when adding MBI or ADAS-DR to models initially only including the covariates.

**Abbreviations:** β = beta coefficient, Aβ = amyloid-β, ADAS-DR = ADAS-cog Delayed Recall, CU = Cognitively Unimpaired, MBI-C = Mild Behavioral Impairment – Checklist, p = p-value, P-tau_181_ = Phosphorylated tau 181, R^2^ = the coefficient of determination, S.E = Standard Error and SUVR = Standard Uptake Value Ratio.

**Supplementary Table 2**. MBI-C domains as predictors of tau deposition in Braak region I-II (imputed data)

| **Model** | **β** | **S.E** | **p** | ^†^**ΔR^2^** | **^*^R^2^** |
| --- | --- | --- | --- | --- | --- |
| Braak I-II ~ MBI-C Drive | 0.033 | 0.019 | 0.077 | 0,0596 | 0.199 |
| Braak I-II ~ MBI-C Affective | 0.023 | 0.010 | **0.020** | 0,1004 | 0.240 |
| Braak I-II ~ MBI-C Control | 0.019 | 0.008 | **0.015** | 0,1093 | 0.249 |
| Braak I-II ~ MBI-C Social | 0.036 | 0.036 | 0.918 | 0,0188 | 0.158 |
| Braak I-II ~ MBI-C Perception | 0.065 | 0.116 | 0.578 | 0,0061 | 0.145 |
| P-tau_181_ ~ MBI-C Drive | 4.617 | 2.560 | 0.078 | 0,06215 | 0.159 |
| P-tau_181_ ~ MBI-C Affective | 3.663 | 1.303 | **0.007** | 0,13745 | 0.235 |
| P-tau_181_ ~ MBI-C Control | 2.676 | 1.047 | **0.014** | 0,11685 | 0.214 |
| P-tau_181_ ~ MBI-C Social | 4.231 | 5.041 | 0.406 | 0,01425 | 0.111 |
| P-tau_181_ ~ MBI-C Perception | 13.115 | 15.892 | 0.414 | 0,01375 | 0.111 |

Multivariate linear regression analyses in 50 CU Aβ-positive subjects using single imputed data for missing MBI-C item responses. The models investigated the association between tau deposition in Braak stage I-II or using CSF P-tau_181_, and the five different MBI-C domains (Drive and motivation [Drive), Affective regulation [Affective], Impulse dyscontrol [Control], Social inappropriateness [Social], Perception and thought [Perception]). Models were adjusted for age, sex, education and WML volume. ^18^F-RO948-PET SUVR in Braak stage I-II or CSF P-tau_181_ was entered as a continuous dependent variable. Continuous MBI-C domain scores, respectively, were entered as the independent variable. ^*^R^2^ for the complete model. ^†^Change in R^2^ when adding MBI or ADAS-DR to models originally only including the covariates.

**Abbreviations:** β = beta coefficient, Aβ = amyloid-β, CU = Cognitively unimpaired, MBI-C = Mild Behavioral Impairment – Checklist, p = p-value, P-tau_181_ = Phosphorylated tau 181, R^2^ = the coefficient of determination, S.E = Standard Error and SUVR = Standard Uptake Value Ratio

**Supplementary references**

1. Jack CR, Jr., Bennett DA, Blennow K, Carrillo MC, Dunn B, Haeberlein SB *et al.* NIA-AA Research Framework: Toward a biological definition of Alzheimer's disease. *Alzheimers Dement* **14**(4)**:** 535-562 (2018).

2. American Psychiatric Association. *Diagnostic and Statistical Manual of Mental Disorders, 5ed*, Vol. 5 (Arlington, VA, 2013).
